# Polyarteritis nodosa with testicular involvement: a rare case report highlighting the role of nuclear imaging and angiography in diagnosis

**DOI:** 10.1093/bjrcr/uaaf032

**Published:** 2025-07-01

**Authors:** Jacob Owens, Gunnar Whealy, Harvey Sekhon, Rustain Morgan, Craig Johnson, Lei Yu

**Affiliations:** College of Medicine, University of Nebraska Medical Center, Omaha, NE 68198, United States; Department of Radiology, University of Nebraska Medical Center, Omaha, NE 68198, United States; Department of Radiology, University of Nebraska Medical Center, Omaha, NE 68198, United States; Department of Radiology, University of Nebraska Medical Center, Omaha, NE 68198, United States; Department of Radiology, University of Nebraska Medical Center, Omaha, NE 68198, United States; Department of Radiology, University of Nebraska Medical Center, Omaha, NE 68198, United States

**Keywords:** vasculitis, polyarteritis nodosa, F-18 FDG PET/CT, angiography

## Abstract

Polyarteritis nodosa (PAN) is a systemic small to medium vessel vasculitis. It is often associated with hepatitis B infection and classically presents with cutaneous, gastrointestinal, or nervous system involvement. We present a case of a 56-year-old male who presented with a chief complaint of painful scrotal swelling. Initial ultrasound demonstrated concern for epididymitis, and the patient was started on appropriate antibiotics without improvement of symptoms, resulting in admission. Due to continued scrotal pain, fevers, and negative infectious work-up, F-18 fluorodeoxyglucose (FDG) PET/CT was obtained, revealing diffuse hypermetabolic activity throughout the medium to small arterial vasculature, concerning for vasculitis. Abdominopelvic angiography confirmed the diagnosis, and the patient was started on steroids with plans to initiate cyclophosphamide. Clinical testicular involvement is a rare presentation of PAN, although it is often seen at autopsy. Previously reported cases have presented with similar scrotal pain and tenderness in addition to constitutional symptoms, as well as treatment with steroids and immunosuppressive agents. While biopsy with histopathology or angiography often serves as the gold standard for the diagnosis of PAN, this case also demonstrates the diagnostic utility of nuclear medicine with F-18 FDG PET/CT. Polyarteritis nodosa typically demonstrates hypermetabolic activity of the small- to medium-sized vasculature on F-18 FDG PET/CT, most often in the lower extremities. With similar findings, this case contributes to reports that show the utility of nuclear imaging in diagnosing vasculitides.

## Introduction

Polyarteritis nodosa (PAN) is a primary systemic necrotizing vasculitis that primarily affects medium to small arteries. Often associated with hepatitis B infection, it may be idiopathic or triggered by other viral infections. The incidence is about 0-1.6 cases per million, with peak incidence in the fifth to sixth decade of life.^1^ This disease classically involves the cutaneous, gastrointestinal, and nervous systems. Testicular involvement is often clinically rare, though it is one of the American College of Rheumatology (ACR) diagnostic criteria for PAN. Diagnosis often comes from clinical, laboratory, histopathologic, and imaging findings, with angiography being the gold standard for imaging diagnosis. We present a case of PAN with testicular involvement, with diagnosis made on the basis of nuclear and angiographic imaging techniques.

## Case

A 56-year-old male with a history of coronary artery disease, myocardial infarct, hypertension, hyperlipidemia, and heart failure presented to the emergency department for painful swelling of his scrotum. Eleven days before admission, the patient reports experiencing generalized myalgias, fatigue, and dysphonia with the development of a large lymph node in the left supraclavicular region. He later developed scrotal swelling 5 days before presentation. Scrotal ultrasound demonstrated signs of epididymitis, and he was started on a 5-day course of levofloxacin. However, his symptoms continued to worsen, developing lower extremity edema and a burning sensation in the thighs. Upon presentation, initial laboratory data demonstrated leukocytosis and elevated procalcitonin and creatine kinase. CT pelvis with contrast revealed fluid in the scrotal sac. Following initial evaluation, the patient was then admitted for further work-up and treatment of suspected epididymo-orchitis.

**Figure 1.**
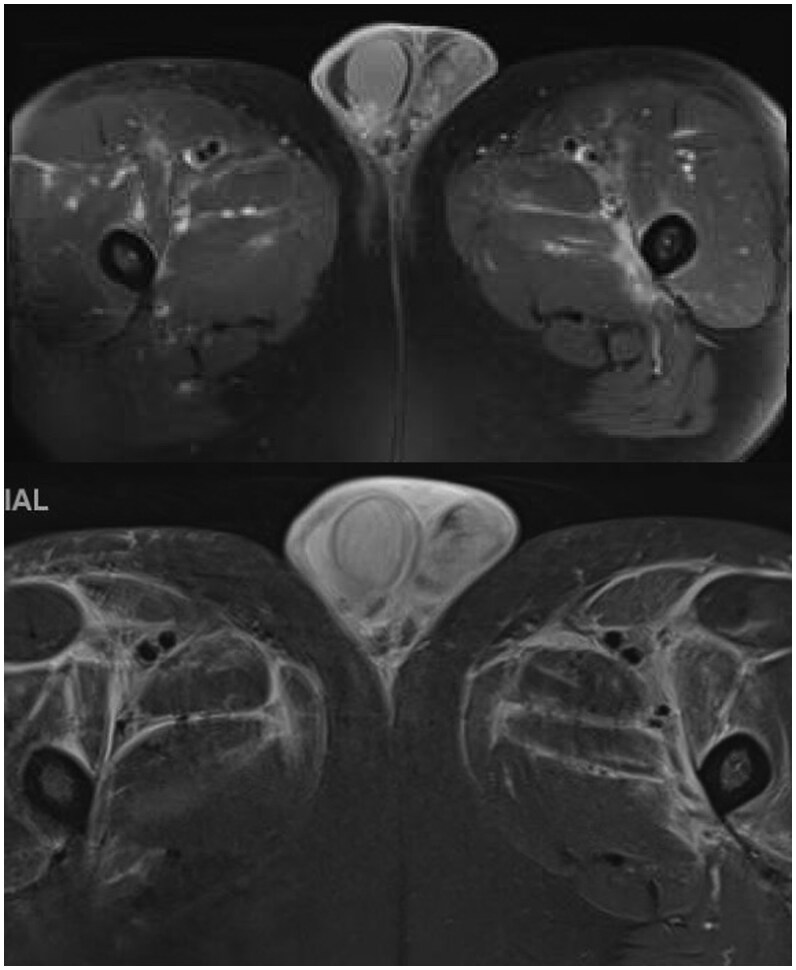
MRI abdomen and pelvis with and without contrast. Scrotal wall edema and small right hydrocele observed on T1 with fat suppression and gadolinium (upper) and STIR (lower). There is marked diffuse superficial, deep edema and muscular enhancement in the bilateral proximal thighs and pelvis, concerning for systemic vascular inflammatory process, myositis, or cellulitis.

**Figure 2.**
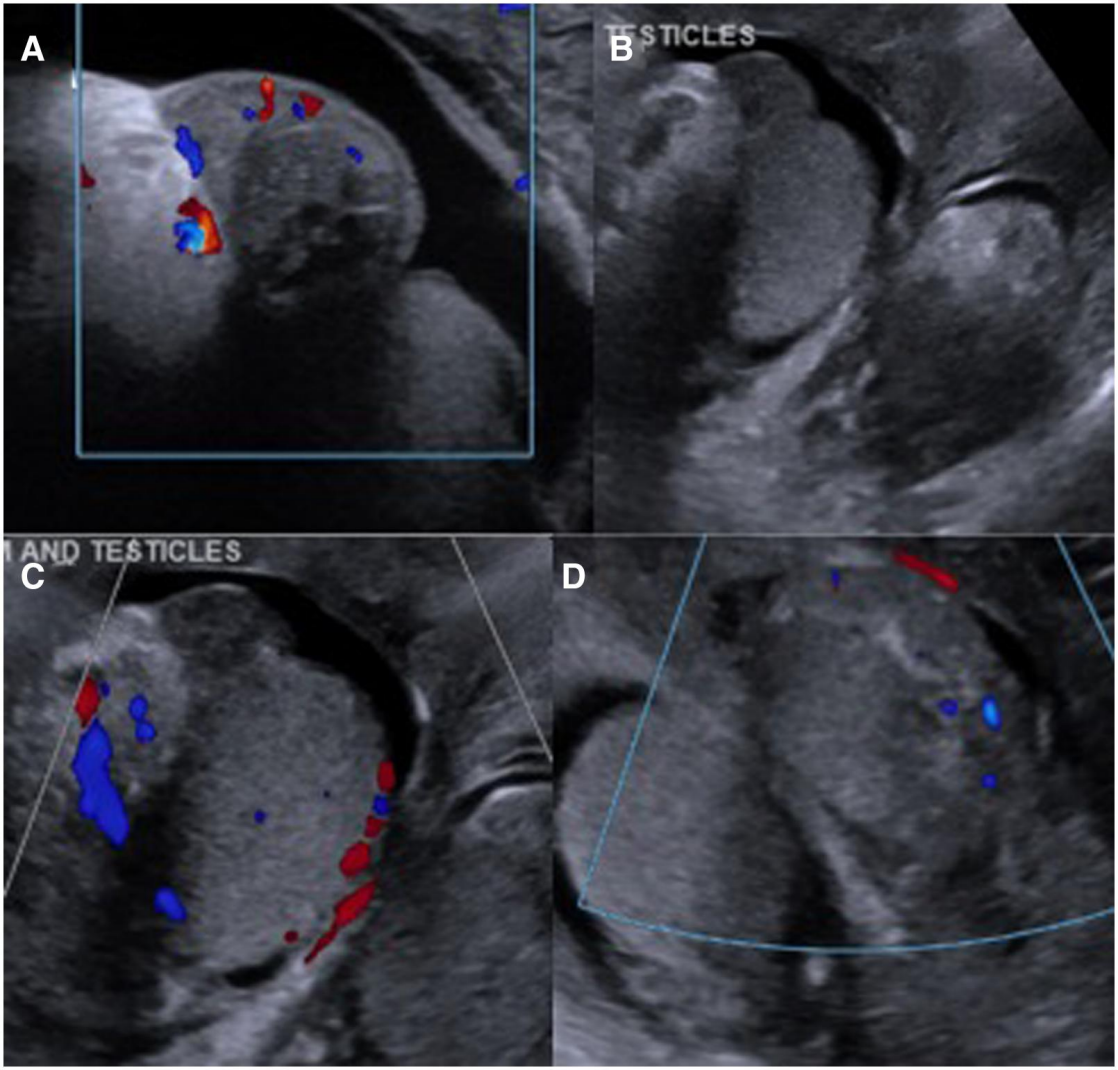
Scrotal Ultrasound. Right testicle measures 4.6 × 3.5 × 3.4 cm and epididymis demonstrates mild heterogenicity and normal flow (A and C). Small right hydrocele (A and B). Left testicle measures 3.2 × 3.1 × 3.7 cm with mild heterogenicity and normal flow (B and D). Scrotal wall edema is also present. Findings are concerning for mild epididymo-orchitis.

During his admission, the patient was placed on multiple courses of antibiotics, including piperacillin-tazobactam, vancomycin, doxycycline, meropenem, and clindamycin. Blood cultures obtained at admission demonstrated no growth. The patient underwent additional imaging with MRI pelvis with contrast, which showed marked diffuse soft tissue edema and enhancement in the proximal thighs, lower pelvis, and scrotal wall, but unremarkable appearances of the testicles and epididymides (Figure 1). Repeat scrotal ultrasound demonstrated normal sized testicles and epididymides with mild heteroechogenicity and scrotal wall edema consistent with mild epididymo-orchitis (Figure 2). After 5 days without clinical improvement, the patient was transferred to our institution for further work-up and treatment.

**Figure 3.**
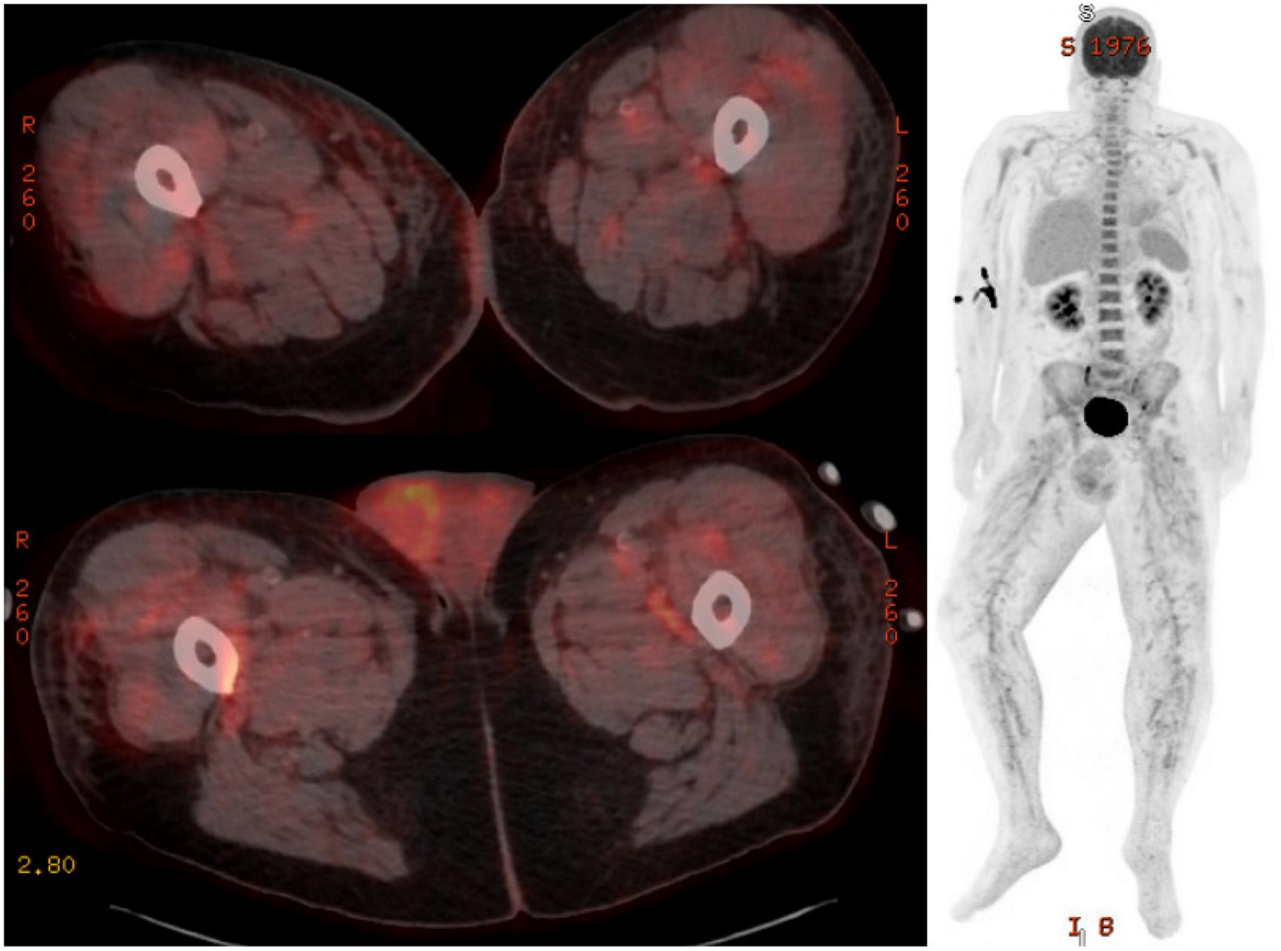
F-18 FDG PET CT scan with axial imaging of the scrotum and proximal thighs (left), as well as maximum intensity projection image (right). Demonstrates diffuse hypermetabolic FDG activity associated predominantly with medium to small arterial vasculature throughout the whole body (right). This is most pronounced in the bilateral lower extremities, with additional enhancement in the bilateral supraclavicular and axillary regions, and pelvis/scrotum.

Following patient transfer, repeat lab work demonstrated increasing leukocytosis and elevated inflammatory markers and ferritin. The patient underwent an extensive infectious (Table 1) work-up, including Karius, which returned negative. Mumps serologies included reactive IgG, but equivocal IgM, with recommendations for repeat testing in 2 weeks. Antinuclear antibodies (ANA) and antineutrophil cytoplasmic antibodies (ANCA) panels were negative. Given the patient’s persistent fevers, F-18 FDG PET/CT was ordered and demonstrated diffuse hypermetabolic activity involving the medium to small arterial vasculature throughout the body with relative sparing of the aorta, concerning for vasculitis (Figure 3). Additionally, there is diffuse hypermetabolic activity within the testicles. Increased bone marrow uptake is consistent with a reactive increase in bone marrow hematopoietic function secondary to patient’s chronic anemia. No biopsy site was identified. Additional CT angiography abdomen and pelvis with contrast showed patent arterial vasculature without evidence of aneurysm. Electromyography (EMG) demonstrated chronic axonal sensorimotor neuropathy and left median mononeuropathy consistent with vasculitis. The patient was started on methylprednisolone with improvement of both symptoms and inflammatory markers.

**Table 1.** Infectious and rheumatological work-up performed before PET/CT and angiography that confirmed patient’s diagnosis of polyarteritis nodosa.

Infectious and rheumatologic work-up	
Diagnostic test	Result
Blood cultures (2 sets)	No growth
Gonorrhea and chlamydia PCR	Negative
Streptococcus pneumoniae antigen	Negative
*Legionella pneumophila* antigen	Negative
Antinuclear antibody screen and Jo-1 antibody	Negative
HIV antigen and antibody panel	Non-reactive
Mumps serologies	IgG reactive IgM 0.92 (equivocal)
EBV serologies	Viral capsid antigen IgM 0.6 (negative) Viral capsid antigen IgG >8.0 (positive) Early antigen-diffuse IgG 2.2 (positive) EBV nuclear antibody IgG >8.0 (positive)
Q fever antibody	Negative
*Brucella abortus* antibody	<1:20 (negative)
Rheumatoid factor	12 (negative)
ANCA panel	Myeloperoxidase antibody 0 (negative) Serine protease 3b antibody 1 (negative) Neutrophil cytoplasmic antibody titer <1:20 (negative)
West Nile serologies	IgG 5.94 (positive) IgM 0.57 (negative)
Lyme serologies	IgG and IgM non-reactive
Tick-borne disease PCR panel	*Babesia*, *Ehrlichia*, and *Anaplasma* spp. not detected
*Francisella tularensis*	1 (negative)
Coccidioides antibody panel	<1:2 (negative)
Karius	Negative

**Figure 4.**
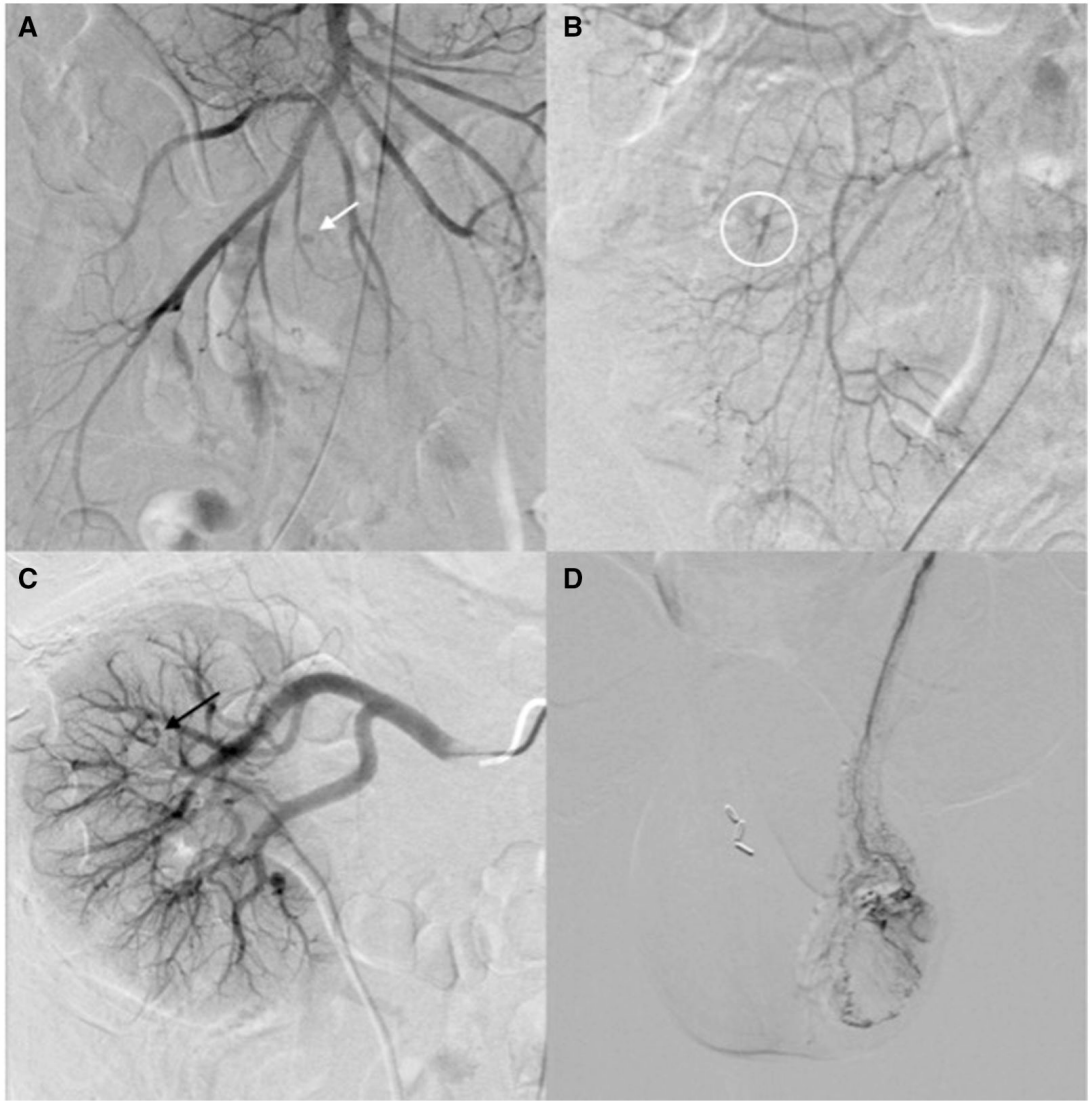
Abdominopelvic angiography of the superior mesenteric artery shows multiple microaneurysms, including a 0.5 cm saccular aneurysm (A, white arrow) and beading appearance (B, white circle) of small vascular branches. Additional angiography of the left renal artery (C) and left gonadal artery (D) demonstrates diffuse involvements of medium and small arterial vasculature with punctate pseudoaneurysms (black arrow), focal dilatation, and stenosis observed consistent with vasculopathy, including polyarteritis nodosa.

The patient ultimately underwent abdominopelvic angiogram (Figure 4). This demonstrated diffuse microaneurysms involving the small- and medium-sized vessels throughout the abdomen and pelvis, consistent with polyarteritis nodosa. Methylprednisolone was continued and eventually switched to oral prednisone. Antibiotics were discontinued. Following patient symptom resolution and continued downtrend in leukocytosis and inflammatory markers, the patient was discharged on 60 mg daily prednisone with plans to start cyclophosphamide infusions for treatment of polyarteritis nodosa.

## Discussion

Polyarteritis nodosa is a medium to small vessel vasculitis, classically affecting the main visceral arteries and their branches.^1^ Although the exact mechanism behind this vasculitis has not been defined, the resulting inflammation of the involved arteries leads to occlusion, stenosis, and/or aneurysms that may produce ischemia and hemorrhage in a variety of organs and tissues.^1^^,^^2^ Cutaneous, neurological, and gastrointestinal manifestations are most common; however, renal, respiratory, cardiac, and genitourinary symptoms may be seen as well.^1^^,^^3^ Constitutional symptoms of fever, malaise, or myalgias are also observed, as in our patient. Management often depends on disease severity, with severe disease being defined as active disease with life or organ-threatening symptom manifestations, such as mononeuritis multiplex, muscle disease, renal disease, or mesenteric ischemia.^4^

Clinical testicular involvement is a relatively rare presentation of PAN. Previous documented cases have similar presentations with testicular pain, scrotal swelling, epididymal pain and swelling, and other constitutional symptoms.^5^^,^^6^ While clinical involvement might be rare, autopsy studies demonstrate much higher rates of involvement in PAN, ranging from 60% to 86%.^6^ If symptomatic, a definitive diagnosis may be made via biopsy when available before relying on imaging modalities.^3^^,^^5^^,^^6^ Recommended treatment for these patients is the same as active PAN with intravenous or oral glucocorticoids and possible immunosuppression depending on the disease severity.^4^

Diagnostic criteria for PAN were originally defined by the 1990 ACR criteria, with later updates in the 1994 and 2012 Chapel Hill Consensus Conferences (Table 2).^1^^,^^7^ Biopsy of symptomatic sites is often utilized to confirm the diagnosis, demonstrating mixed inflammatory infiltrates with granulocytes and lymphocytes in the arterial wall. Combined muscle and nerve biopsies have also been shown to have a higher diagnostic yield compared to isolated muscle biopsies.^1^ In our case, our patient did not undergo biopsy as no definitive appropriate biopsy site was identified on PET/CT, with EMG and abdominopelvic angiogram being relied on instead. Angiography is the most sensitive imaging modality and is recommended when biopsy is unavailable, classically demonstrating multiple 2–5 mm saccular or fusiform aneurysms commonly located at arterial branch points.^1–4^^,^^8^ Angiogram in our case demonstrated these findings in the distal superior mesenteric artery. The kidney is the most frequently involved organ seen on angiography, while mesenteric arteries tend to have smaller and fewer lesions.^8^ Outside of angiography, other imaging techniques, such as CT or MRI, have generally demonstrated only the complications of this disease, with some additional research into the applications of nuclear imaging.

**Table 2.** 1990 ACR diagnostic criteria. Criteria were later expanded on by the Chapel Hill Consensus Conference to further differentiate from microscopic polyangiitis and ANCA-positive vasculitides.^7^

1990 ACR diagnostic criteria
Weight loss of greater than 4 kg
Livedo reticularis
Testicular pain or tenderness not due to infection, trauma, or other causes
Diffuse myalgias of leg muscles
Mononeuropathy, multiple mononeuropathies, or polyneuropathy
Diastolic blood pressure >90 mmHg
Blood urea nitrogen elevation (>40 mg/dL) or creatinine elevation (>1.5 mg/dL)
Positive hepatitis B antigen or surface antibody in serum
Arteriogram demonstrating aneurysms or occlusions of the visceral arteries not due to noninflammatory causes
Biopsy of small- to medium-sized arteries demonstrating granulocytes

In our case, F-18 FDG PET/CT was utilized due to persistent fevers and demonstrated diffuse FDG uptake within medium-sized arteries, concerning for vasculitis. Previous utility for FDG-18 PET/CT has been demonstrated for vasculitis in general, with positive findings in most patients.^9^ In patients with PAN, PET/CT has shown diffuse involvement of the medium and small vessels in the lower extremities, often distributed in reticular or linear patterns.^9^^,^^10^ Other findings have included moderate bone marrow activation, hypersplenism, and hypermetabolism of larger visceral arteries, such as the humeral, femoral, and popliteal arteries.^10^ However, patients who have been on corticosteroids have had negative nuclear imaging findings, even if they met ACR criteria.^10^ With similar findings observed in our case, PET/CT may serve as an additional and less invasive imaging modality to aid in diagnosis.

## Conclusion

Here, we present a rare case of newly diagnosed polyarteritis nodosa with testicular involvement being the main clinical manifestation. While this case utilizes the gold standard in imaging techniques for diagnosis, angiography, it is also unique in that the work-up and diagnosis was particularly aided by F-18 FDG PET/CT. These findings on PET/CT contributed to the work-up that led to a definitive diagnosis with angiography, corroborating previous research demonstrating the utility of nuclear imaging in diagnosing vasculitides.

## Learning points

- PAN is classically diagnosed by either tissue biopsy or angiography following clinical suspicion.
- F-18 FDG PET/CT demonstrates diffuse involvement of the medium and small vessels in the lower extremities of patients with PAN, often distributed in reticular or linear patterns.
- Nuclear medicine imaging with F-18 FDG PET/CT demonstrates utility in diagnosing vasculitides, including PAN.
